# Multi-omics analysis reveals the mechanism of adventitious roots formation in peach green branch cuttings

**DOI:** 10.3389/fpls.2025.1740503

**Published:** 2026-01-05

**Authors:** Fan Zhang, Jiaxuan Ren, Chenbing Wang

**Affiliations:** Institute of Forestry, Fruits and Floriculture, Gansu Academy of Agricultural Sciences, Lanzhou, China

**Keywords:** adventitious root, indole-3-butyric acid, metabolomics analysis, proteomics analysis, *Prunus persica*, transcriptome analysis

## Abstract

**Introduction:**

During asexual propagation of peach rootstocks, adventitious root (AR) formation is influenced by multiple factors, with exogenous hormone application being a key strategy. However, the molecular mechanisms underlying AR formation remain incompletely understood.

**Methods:**

In this study, we treated ‘GF677’ peach rootstocks with 200 mg/L indole-3-butyric acid (IBA) and analyzed the molecular mechanism of AR formation using transcriptomic, proteomic, and metabolomic analysis.

**Results:**

By detecting the rooting rate and the ratio of indole-3-acetic acid-to-cytokinin (IAA/CTK), we confirmed that 21 days of treatment with 200 mg/L IBA represented the critical time point for AR formation in ‘GF677’ rootstocks. The transcriptomic analysis identified 3,305 differentially expressed genes (DEGs), the proteomic analysis revealed 1,221 differentially expressed proteins (DEPs), and the metabolomic profiling screened key metabolites, including 10 hormone-associated differential metabolites. Furthermore, KEGG pathway enrichment analysis across the multi-omics datasets identified two core co-enriched pathways: plant hormone signal transduction and biosynthesis of secondary metabolites. Through multi-omics analysis, we identified DEGs and found that genes related to auxin synthesis pathways (*GRETCHEN HAGEN3* (*GH3*), *PIN-FORMED* (*PIN*), *SMALL AUXIN-UP RNA* (*SAUR*), *AUX/IAAs*, and *IAA-Leucine Resistant 1* (*ILR1*)), CTK synthesis pathways (*Cytokinin Oxidase/Dehydrogenase 7* (*CKX7*), *Zeatin O-Glucosyltransferase* (*ZOG*), and *Isopentenyltransferase 3* (*IPT3*)), transcription factors related to plant hormones (*Auxin Response Factor* (*ARF*), *Myeloblastosis* (*MYB*), *Myelocytomatosis* (*MYC*), *Basic Helix-Loop-Helix* (*bHLH*), *GAI-RGA-SCR* (*GRAS*), *APETALA2/Ethylene Responsive Factor* (*AP2/ERF*), and *Basic Leucine Zipper* (*bZIP*)), and phenylpropanoid biosynthesis pathway (*4-Coumarate-CoA Ligase* (*4CL*), *Phenylalanine ammonia-lyase* (*PAL*), *cinnamate-4-hydroxylase* (*C4H*), and *Flavonol synthase* (*FLS*)) were significantly affected by IBA treatment. Quantitative real-time PCR (qRT-PCR) validation of eight key DEGs confirmed transcriptomic reliability.

**Conclusion:**

These findings suggest that IBA promotes AR formation in peach rootstocks by modulating plant hormone levels and enhancing phenylpropanoid biosynthesis.

## 1 Introduction

The peach rootstock industry is undergoing a transition, shifting from traditional seedling rootstocks to self-rooted rootstocks (Nicolao et al., 2025). In accordance with the national policy of non-grain cultivation, fruit tree cultivation needs to expand into mountainous areas, thereby necessitating the strengthening of improved variety breeding systems. However, peach production areas in Gansu Province are mostly located in mountainous and other infertile regions, characterized by drought and cold conditions, low soil organic matter content, saline-alkali soil, and continuous cropping. These factors severely restrict the development of the peach industry (Liu et al., 2010). However, due to the immaturity of low-cost clonal propagation technology for peach rootstocks and the inherent difficulty of rooting in peach, addressing the challenges in peach rootstock clonal propagation is critical for advancing modern sustainable fruit farming (Okie and Nyczepir, 2004; Thakur et al., 2023). Additionally, the rooting process in peach rootstock clonal propagation is influenced by multiple factors. Among these, promoting adventitious roots (ARs) formation through exogenous hormone-induced changes in endogenous hormone levels represents one of the primary strategies (Timm et al., 2015; Li S. W, et al., 2016; Quan et al., 2017). Therefore, identifying the key factors that influence the formation of ARs in peach rootstocks clonal propagation and delving into the mechanisms through which these factors exert their influence are essential for enhancing the AR production capacity of peach rootstocks.

The formation of AR is closely related to tissue dedifferentiation and is regulated by exogenous plant hormones. Auxin has been proven to be an effective inducer of ARs in numerous plant species, potentially interacting with other endogenous factors or environmental stimuli during this process (Sorin et al., 2006). Horticultural and agricultural practices have demonstrated that the exogenous application of auxin significantly promotes AR formation in various plant species. Indole-3-butyric acid (IBA) is widely used globally as a hormone to promote rooting due to its higher stability and efficiency compared to indole-3-acetic acid (IAA). In cells, IBA can be converted into IAA, acting as a sustained-release source of IAA (Ludwig-Müller et al., 2005; Pacurar et al., 2014). Although auxin plays a central role in promoting cell dedifferentiation and root primordium formation, the specific mechanisms underlying its action remain largely unknown.

Over the past few decades, the physiological and biochemical changes during the complex process of in vitro AR development have been extensively studied. However, the underlying molecular mechanisms of this process still require in-depth exploration. In recent years, various molecular and genetic approaches have been employed to identify genes that regulate AR development in *Arabidopsis thaliana* and other plant species. Among the identified genes, several gene families involved in the auxin signaling pathway have been demonstrated to control AR formation in various plants, such as *Auxin Response Factor* (*ARF*) family (Sun et al., 2025; Li, 2021), *GRETCHEN HAGEN3* (*GH3*) family (Staswick et al., 2005), *SMALL AUXIN-UP RNA* (*SAUR*) family (Wang et al., 2024), auxin efflux carrier genes *PIN-FORMED (PIN)* (Blilou et al., 2005), and auxin-responsive genes *AUX/IAAs* (Lakehal et al., 2019). Conversely, cytokinin (CTK) may antagonize auxin activity and inhibit the development of ARs in various plant species (Ramírez-Carvajal et al., 2009; Kitomi et al., 2011; Werner et al., 2003). Ai et al. (2023) observed significant changes in the expression of transcription factors such as APETALA2/Ethylene Responsive Factor (*AP2/ERF*), Basic Helix-Loop-Helix (*bHLH*), *WRKY*, NAM-ATAF1/2-CUC2 (*NAC*), Myeloblastosis (*MYB*), Cys2-His2 (*C2H2*), Basic Leucine Zipper (*bZIP*), and GAI-RGA-SCR (*GRAS*) during AR formation in *Medicago sativa*. Nonetheless, the gene expression profile in response to auxin-induced AR formation in peach rootstocks remains uncharacterized.

Previous studies have demonstrated that during AR formation in Garnem and ‘GF677’ rootstocks, which exhibit varying rooting capacities respectively, endogenous hormone homeostasis undergoes significant dynamic changes in vitro microcuttings, with key roles played by metabolites such as IAA and IAA-aspartic acid (Justamante et al., 2022). In the context of IBA-induced AR formation in dwarf apple rootstocks, a total of 3,355 differentially expressed proteins (DEPs) have been identified, primarily associated with carbohydrate metabolism and energy production, protein homeostasis, reactive oxygen species and nitric oxide signaling, cell wall remodeling, and plant hormone signaling pathways (Lei et al., 2018). Moreover, differentially expressed genes (DEGs) and metabolites associated with AR formation in *Eucommia ulmoides* are predominantly enriched in phenylpropanoid, flavonoid, and isoflavonoid biosynthesis pathways (Du et al., 2024). However, research on the auxin-responsive mechanisms underlying AR formation in peach rootstocks remains limited, particularly regarding the comprehensive molecular mechanisms of IBA-induced AR formation. A systematic investigation integrating transcriptomics, proteomics, and metabolomics is still lacking, making this an essential focus for future studies.

Based on previous research conducted by our group (Zhang et al., 2013; Zhang et al., 2025), we found that the application of 200 mg/L IBA was most effective in promoting rooting in peach rootstocks. In this study, peach rootstocks were treated with 200 mg/L IBA for 0, 3, 6, 9, 12, 18, and 21 days, respectively. Subsequently, the contents of IAA and CTK in the phloem were quantified, and the critical point for AR formation in ‘GF677’ was determined through analysis of the rooting rate and IAA/CTK ratio. Finally, transcriptomic, proteomic, and metabolomic analyses were employed to assess the potential molecular mechanisms underlying the effect of IBA on AR formation in peach rootstocks. This study aims to provide both practical and theoretical insights into the propagation and rooting mechanisms of peach cuttings, with potential applications for other woody plant species.

## 2 Materials and methods

### 2.1 Plant material and sample preparation

An experimental study was conducted to investigate the propagation of greenwood cutting from the peach rootstock ‘GF677’. Firstly, branches of uniform thickness were carefully selected and rinsed with flowing water for one hour to clean their surfaces before cutting. Subsequently, the branches were cut into segments measuring 6–8 cm in length, ensuring that each segment contained 4–5 buds. The basal incision was made 0.5 cm away from the first bud to create a bevel, while the top flower buds were located approximately 1 cm from the incision. The experimental design comprised two groups: the experimental group underwent induction treatment with a 200 mg/L IBA (analytical grade, Sigma, USA) solution; the control group was treated with water. After treatment, the cuttings were inserted into the nursery bed at a consistent depth of 3 cm. Peach rootstock propagation frequently encounters challenges such as poor rooting capacity. AR formation entails the perception of exogenous signals and their integration with internal signalling cascades, constituting a highly complex process of non-root organogenesis. Given that root primordia in non-root organs predominantly develop within the phloem of cuttings, the phloem was selected as the target tissue for sampling. Subsequently, phloem samples were collected from the area 2 cm above the base of the cuttings at 0, 3, 6, 9, 12, 18 and 21 days after cutting. After the epidermis at the base was removed with a scalpel, the phloem was scraped off while care was taken to avoid contamination with the xylem. All samples were immediately flash-frozen in liquid nitrogen and stored for subsequent analysis of hormone content in the cuttings, aiming to investigate the impact of IBA treatment on hormonal dynamics during the rooting process of ‘GF677’ peach rootstock. Furthermore, peach rootstock samples treated with water for 21 days (control 21) and with 200 mg/L IBA for 21 days (T21) were selected for comprehensive transcriptomic, proteomic, and targeted metabolomic analyses in this study.

### 2.2 Measurement of endogenous hormone content

For each replicate, 100 cuttings were statistically analyzed. A total of three biological replicates were set up. The rooting rate was calculated based on the number of rooted cuttings. Rooting rate = Number of rooted cuttings/100 × 100%. According to the kit instruction manual provided by Shanghai Enzyme-Linked Biotechnology, the Enzyme-Linked Immunosorbent Assay (ELISA, ml022829) method was utilized to accurately quantify the levels of IAA and CTK. Three biological replicates were set up for each time point, and the results are expressed as the average of three biological replicates, with graphs plotted using Graphpad Prism 10.0 software.

### 2.3 Transcriptome sequencing

In this experiment, phloem samples from peach branches treated with control and IBA for 21 days were sent to Metware Biotechnology Co., Ltd. (Wuhan, China) for RNA extraction, quality assessment, library preparation, RNA-Seq, and data analysis. Each treatment group included three replicates. RNA was extracted by ethanol precipitation and CTAB-PBIOZOL. mRNA enrichment was achieved using Oligo (dT) magnetic beads, followed by fragmentation with a dedicated fragmentation buffer. Subsequently, the first-strand cDNA was synthesized using random hexamer primers, followed by the synthesis of the second-strand. After purification using DNA magnetic beads, end-repair, A-tailing, and adapter ligation were performed. Finally, the cDNA library for sequencing was enriched by PCR.

After library construction, the library was initially quantified using the Qubit fluorometric method. Subsequently, the insert size of the library was assessed using a fragment analyzer, and only libraries meeting the expected insert size criteria were used for further experiments. After passing quality control checks, the libraries were pooled according to their target sequencing depth and sequenced on the Illumina platform. The raw sequencing data were filtered to obtain high-quality clean data, which were then aligned with the reference genome using HISAT2 to generate Mapped Data (Kim et al., 2015). The transcript or gene expression levels were quantified using Fragments Per Kilobase Million (FPKM). Differential expression analysis was performed using DESeq2/edgeR, and the statistical summary included the total number of DEGs, as well as the counts of upregulated and downregulated genes in each group. Genes with a |log_2_Fold Change (FC)| ≥ 1 and a False Discovery Rate (FDR) < 0.05 were identified as significantly differentially expressed. Finally, functional enrichment analysis of the DEGs was conducted based on Gene Ontology (GO) and Kyoto Encyclopedia of Genes and Genomes (KEGG) pathways. The FPKM value heatmap of DEGs were plotted by https://www.bioinformatics.com.cn, an online platform for data analysis and visualization (Tang et al., 2023).

### 2.4 Proteomics analysis

Proteins were extracted from the phloem of peach cutting branches treated with control and IBA using the cold acetone method. The samples were ground into powder in liquid nitrogen and then dissolved in 2 mL L3 lysis buffer (1% SDS, 100 mmol/L Tris-HCl, 7 mol/L urea, 2 mol/L thiourea, 1 mmol/L PMSF, and 2 mmol/L EDTA). After being vortexed and mixed, the samples were sonicated on ice for 10 minutes and then centrifuged to obtain the supernatant as the protein extract. Four volumes of cold acetone were added to the protein solution, and the mixture was precipitated at -20 °C overnight. After centrifugation at 4 °C, the precipitate was retained and washed three times with cold acetone. The precipitate was then redissolved in 8 mol/L urea, and the protein concentration was measured using the BCA protein assay kit (P0011, Beyotime, Shanghai).

Take 100 μg protein solution and dilute it to a final volume of 200 μL with 8 mol/L urea. DTT was added, and the mixture was incubated for 45 minutes to allow the reduction reaction to proceed. Subsequently, iodoacetamide was added to achieve a final concentration of 11 mmol/L for alkylation at room temperature. Enzymatic digestion was performed overnight using 25 mmol/L ammonium bicarbonate buffer and 2 μL trypsin (Promega, USA). After digestion, adjust the pH of the solution to 2–3 using 20% trifluoroacetic acid (TFA). The solution was then desalted using C18 resin (Millipore, Billerica, MA). Finally, the peptide concentration was quantified using the Pierce™ quantitative peptide assay kit (A65453, Thermo Fisher Scientific, USA) according to the manufacturer’s instructions and compared with standard peptides.

The samples were separated using the Vanquish Neo UHPLC nanoliter liquid chromatography system, followed by further chromatographic separation performed on the nanoliter-flow Vanquish Neo system (Thermo Fisher Scientific, USA). The separated samples were then analyzed via Data-Independent Acquisition (DIA) on the Orbitrap Astral high-resolution mass spectrometer (Thermo Scientific Scientific, USA). In this study, DIA-NN software (v1.8.1) was employed for qualitative and quantitative analysis of the mass spectrometry data, with peptide and protein abundances calculated using the iBAQ method. Protein functional annotation was conducted based on the GO and KEGG databases, and a detailed investigation was carried out on the significantly enriched GO functions and KEGG pathways associated with DEPs with *P* ≤ 0.05.

### 2.5 Metabolomics analysis

For this assay, 50 mg of ground sample was accurately weighed in liquid nitrogen and subsequently dissolved in 1 mL methanol/water/formic acid solution (15:4:1, V/V/V). After centrifugation and concentration, the extract was reconstituted with an 80% methanol solution and transferred to sample vials for liquid chromatography-tandem mass spectrometry (LC-MS/MS) analysis (Li Y, et al., 2016). The sample extracts were analyzed using a UPLC-ESI-MS/MS system (UPLC, ExionLC™ AD; MS, QTRAP^®^ 6500+) (Xiao et al., 2018; Šimura et al., 2018).

The mass spectrometry data were preprocessed using Analyst 1.6.3 software, followed by an in-depth analysis of the preprocessed data with MultiQuant 3.0.3 software. During the preprocessing, the retention time and peak shape information of standard substances were referenced to perform integral correction on the chromatographic peaks of analytes across different samples, thereby ensuring the accuracy of both qualitative and quantitative analyses. In the data analysis phase, metabolites with a FC ≥ 2 or ≤ 0.5 were identified as significantly differential metabolites. To further elucidate the biological significance of these differential metabolites, we mapped them to the KEGG metabolic pathways for pathway enrichment analysis.

### 2.6 Quantitative real-time PCR analysis

Through integrated multi-omics analysis, we identified several significant DEGs in cuttings at 21 days post-IBA treatment. These DEGs encompassed *GH3* and *IAA* in auxin biosynthesis, *Isopentenyltransferase 3* (*IPT3*) in cytokinin biosynthesis, the plant hormone-related transcription factors *MYB*, *bHLH*, *GRAS*, and *bZIP*, as well as *cinnamate-4-hydroxylase* (*C4H*) involved in phenylpropanoid biosynthesis. Based on this, we chose eight genes exhibiting substantial differences in expression levels for qRT-PCR validation, thereby confirming the reliability of the transcriptomic findings. The RNA used for quantitative analysis and transcriptome sequencing was derived from the same batch of samples. Total RNA was meticulously isolated from the test samples, with particular attention paid to maintaining its integrity and purity. First-strand cDNA synthesis was performed using the Hifair^®^ III 1st Strand cDNA Synthesis SuperMix for qPCR (11141es60, Yeasen, China). Genomic DNA digestion was performed by preparing the reaction mixture on ice, consisting of 3 μL of 5× gDNA Digester Mix, 1 μg of total RNA, and RNase-free water to a final volume of 15 μL. The mixture was incubated at 42 °C for 2 minutes, followed by storage at 4 °C. Subsequently, 15 μL of the digested reaction was combined with 5 μL of 4× SuperMix (final volume 20 μL), and reverse transcription was conducted under the following thermal cycling conditions: 25 °C for 5 minutes, 55 °C for 15 minutes, and 85 °C for 5 minutes. The resulting cDNA was stored at 4 °C for subsequent analysis. For qPCR, the Hieff^®^ qPCR SYBR Green Master Mix (11201ES03, Yeasen, China) was used on an AB-7500 real-time PCR system. After thawing and thoroughly mixing the reagents and primers on ice, a 20 μL reaction system was prepared containing 10 μL of Master Mix, 0.4 μL each of 10 μmol/L forward and reverse primers, 100–200 ng of cDNA, and nuclease-free water. The qRT-PCR program included an initial denaturation step at 95 °C for 5 minutes, followed by 40 cycles of 95 °C for 10 seconds and 60 °C for 30 seconds, and a final melting curve analysis according to the instrument’s default settings. *Actin* was used as an internal reference gene. Samples at each time point were set up with three biological replicates. The expression levels were analyzed using the 2^-ΔΔCt^ method. The primer sequences used in this study are listed in **Supplementary Table S1**.

### 2.7 Statistical analysis

This study utilized SPSS 18.0 software and conducted a one-way analysis of variance (ANOVA) to statistically evaluate the relative expression levels of DEGs. The qRT-PCR results were expressed as mean ± SE of three biological replicates and were plotted as bar graphs using Origin 2018 software. In the analysis, differences with a *P*-value less than 0.05 were considered statistically significant. Before conducting the variance analysis, the arcsine square root transformation is performed on the root growth rate data to ensure the homogeneity of variances and meet the assumptions of the parametric test.

## 3 Results

### 3.1 The impact of IBA on the rooting rate and endogenous hormone levels during AR formation in peach rootstocks

**Figure 1:**
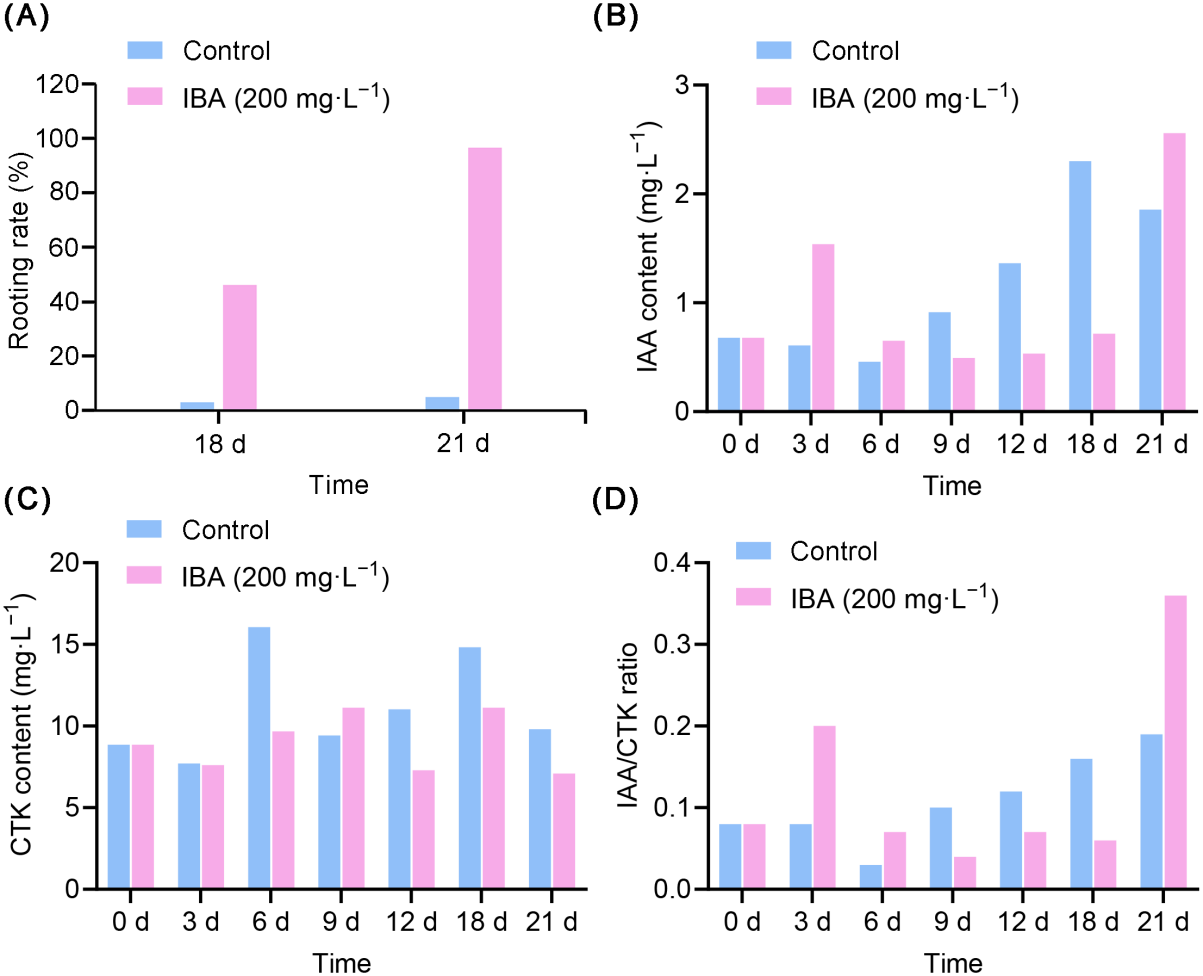
The effects of IBA on the regulation of rooting rate and endogenous hormone levels during AR formation in peach rootstock. **(A)** Variations in the rooting rate in the peach rootstock ‘GF677’ after treatment with 200 mg/L IBA for 18 days and 21 days. **(B)** Variations in the IAA levels in the peach rootstock ‘GF677’ after treatment with 200 mg/L IBA. **(C)** Variations in the CTK levels in the peach rootstock after treatment with IBA. **(D)** The effect of IBA treatment on the changes of IAA/CTK ratio in peach rootstock ‘GF677’. Among them, 0 d represents the experimental materials treated with clear water. 3, 6, 9, 12, 18, and 21 days respectively represent the experimental materials treated with 200 mg/L IBA for 3, 6, 9, 12, 18 and 21 days.

This study aims to investigate the effect of 200 mg/L IBA on AR formation in peach rootstocks and to determine the optimal time point for promoting root growth. To achieve this objective, we conducted an in-depth analysis of the dynamic changes in rooting rate, IAA and CTK levels, thereby elucidating the physiological mechanisms underlying AR formation. The root formation of the peach rootstock ‘GF677’ began 18th day after the cuttings were treated with IBA. Therefore, we calculated the rooting rates on the 18th and 21st day (**Supplementary Figure S1**). Compared with the control group, the rooting rate of the IBA-treated plants reached 46% on the 18th day, and reached the highest level of 96.48% on the 21st day (**Figure 1A**). Compared with the control group, the IAA levels in peach rootstocks increased on the 3rd and 6th days after IBA treatment. However, on the 9th day, the IAA levels began to gradually decrease, continuing to decline on the 12th and 18th days. By the 21st day, the IAA level had risen again (**Figure 1B**). Compared with the control group, IBA treatment reduced the CTK content in peach rootstocks by 60.26% on the 6th day. Subsequently, the CTK levels exhibited a downward trend on the 12th, 18th, and 21st days (**Figure 1C**). In summary, these data indicate that IBA affects the levels of endogenous hormones (IAA and CTK) in peach rootstocks.

The level of IAA plays a pivotal role in plant root development, and its ratio relative to CTK is closely related to AR formation. Therefore, to determine the impact of IBA treatment on the balance of endogenous hormones in peach rootstocks, we observed the variations in the IAA/CTK ratio (**Figure 1D**). Compared with the control group, the IAA/CTK ratio peaked at 21 days after IBA treatment. Based on this observation, we preliminarily confirmed that treatment with 200 mg/L IBA for 21 days represented the critical time point for AR formation in ‘GF677’. Therefore, we selected the samples treated for 21 days and further conducted transcriptomics, proteomic, and metabolomic analyses. This multi-omics approach aims to uncover the molecular regulatory mechanisms underlying AR formation in the peach rootstock ‘GF677’.

### 3.2 Transcriptomic differences between control 21 and T21

**Figure 2:**
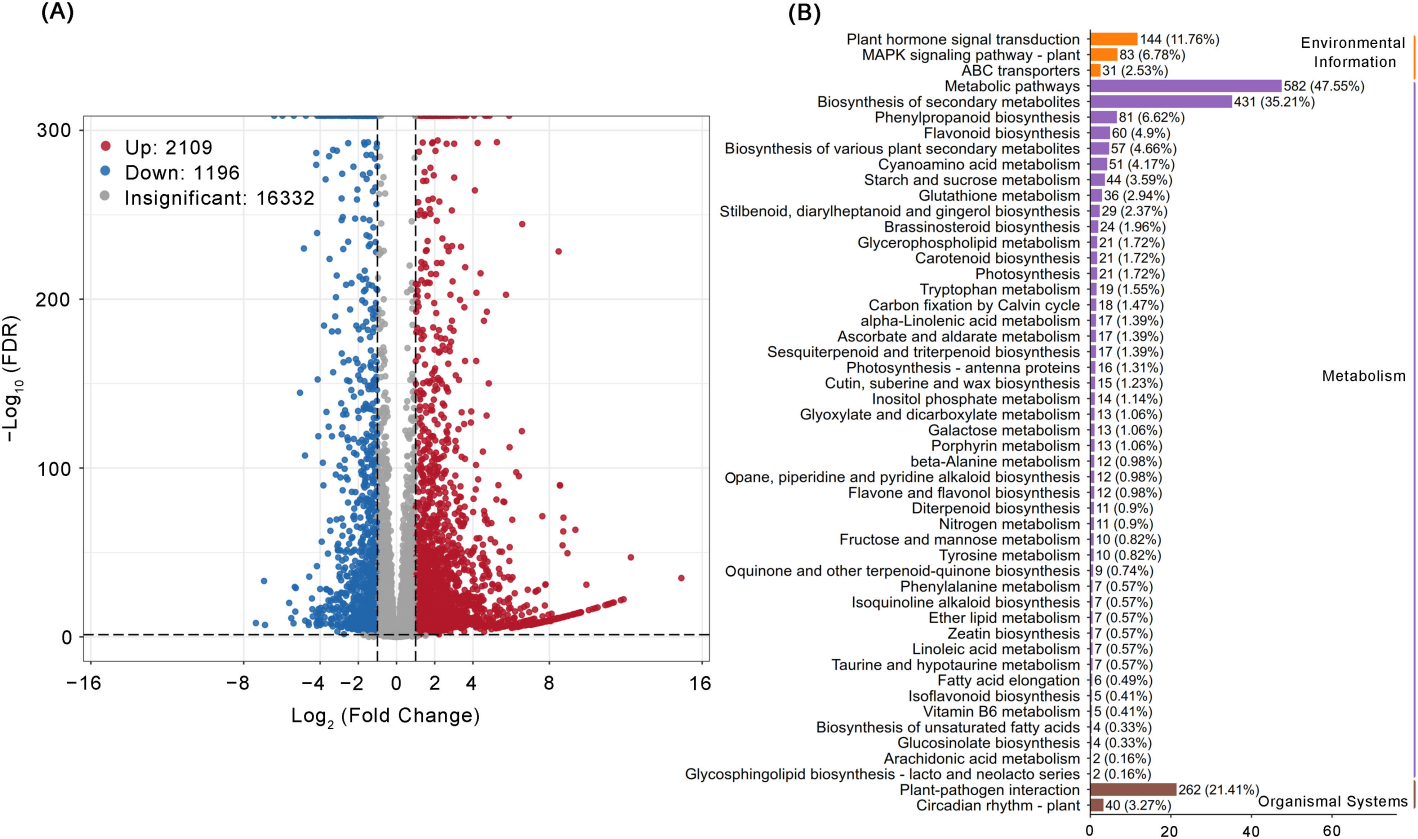
The effect of 200 mg/L IBA treatment on the transcriptome of ARs generated in peach rootstock ‘GF677’ after 21 days. **(A)** Volcano plot of DEGs under control and IBA treatment; the x-axis represents the FC in gene expression, and the y-axis represents the significance level of DEGs. **(B)** The KEGG enrichment bar chart of DEGs shows the top 50 pathways with the most significant enrichment.

Through transcriptome analysis of peach rootstocks treated with and without IBA for 21 days, the molecular mechanism of AR formation was deeply explored. In this study, six samples (including three biological replicates at each sample) were used to construct cDNA libraries, and a total of 54.56 Gb of clean data was generated. The sequencing results showed that the overall sequencing error rate was extremely low (only 0.01%), the Q20 value ranged from 99.03% to 99.11%, the Q30 value ranged from 96.99% to 97.23%, and the GC content was between 45.05% and 45.38%. These indicators collectively indicated the excellent quality of the RNA-Seq and library preparation in this study (**Supplementary Table S2**). DEGs in peach rootstocks treated with IBA were identified using DESeq2/edgeR (with the screening criteria of |log_2_FC| ≥1 and FDR < 0.05). The volcano plot results showed that there were 3,305 DEGs between T21 and contrl 21 (2,109 up-regulated and 1,196 down-regulated) (**Figure 2A**). Furthermore, KEGG enrichment analysis based on transcriptome analysis indicated that DEGs were mainly concentrated in plant hormone signal transduction, amino acid metabolism, starch and sucrose metabolism, phenylpropanoid biosynthesis, carbon fixation by Calvin cycle, and ascorbate and aldarate metabolism (**Figure 2B**). These results were consistent with the findings from proteomic data.

### 3.3 Proteomic differences between control 21 and T21

**Figure 3:**
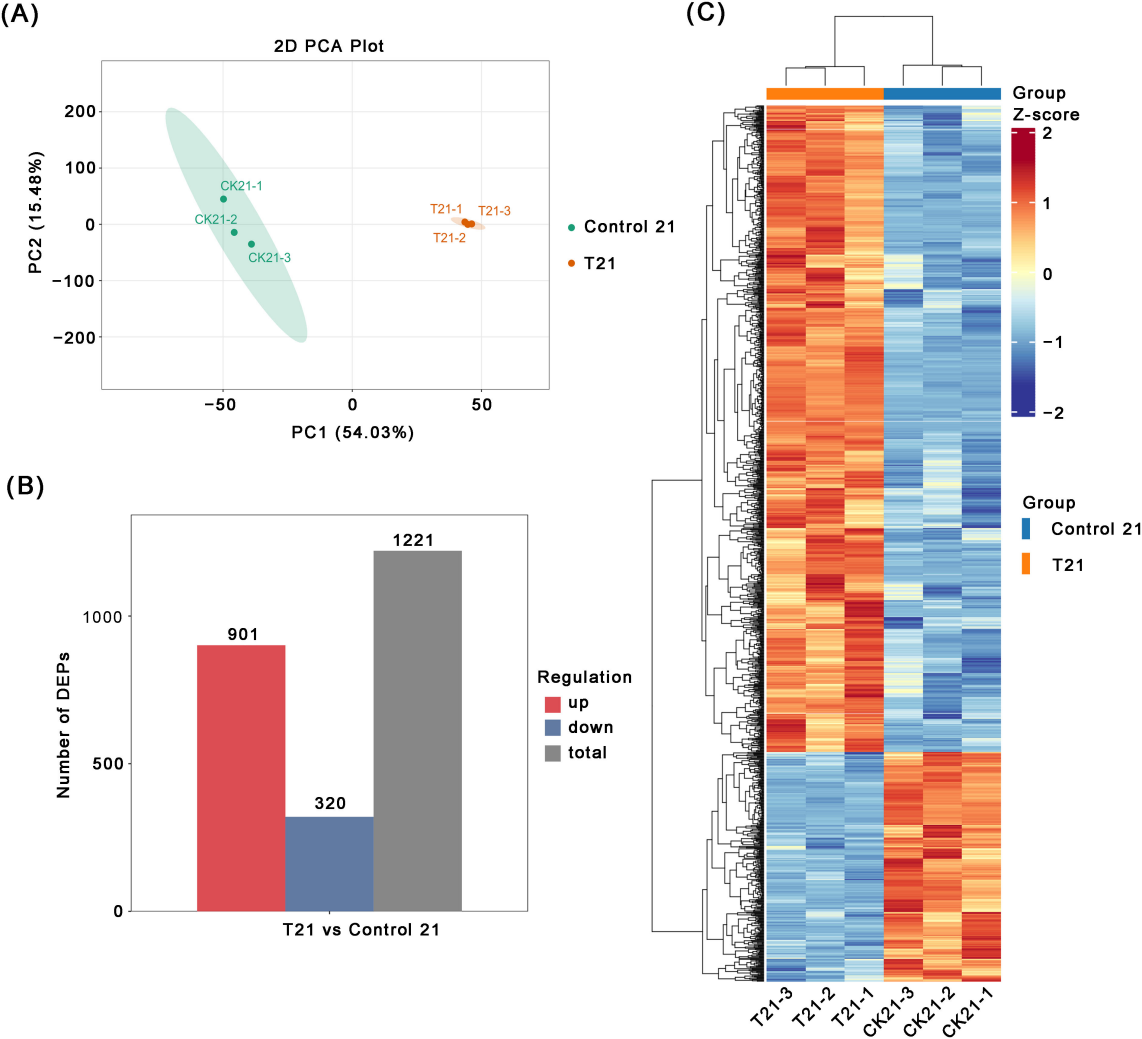
Preliminary analysis of proteomic data. **(A)** Principal component analysis among samples of each group. **(B)** The statistical results of DEPs under the control and IBA treatment conditions. The vertical axis represents the number of DEPs. Red and blue denote up-regulated and down-regulated genes, respectively, whereas grey indicates the total number of genes. **(C)** Heatmap of DEPs between the control and IBA treatment groups. The vertical axis represents the differential proteins, and the horizontal axis represents the samples. Red indicates a high Z-score, reflecting elevated indicator levels in the T21 group, whereas blue indicates a low Z-score, corresponding to reduced indicator levels in the control 21 group. Intermediate transition levels are represented by yellow, white, and light blue.

To identify key proteins involved in the AR formation of peach rootstock ‘GF677’ treated with IBA, we employed proteomic technology. After the mass spectrometry data collection was completed, these data underwent a strict quality control assessment. The assessment included analyses of peptide length distribution and peptide quantities distribution, all of which confirmed that our data met the quality control standards (**Supplementary Figure S2**). The PCA analysis revealed that samples from different treatment groups were distinctly separated in the PCA plot, while samples within the same group clustered together, thereby effectively confirming the reliability of the biological replicates (**Figure 3A**). Subsequently, differential protein expression analysis was conducted. Compared with the control group, a total of 1,221 DEPs were identified in the IBA-treated group, with 901 upregulated and 320 downregulated proteins, respectively (**Figure 3B**). The results of differential protein screening are presented in **Supplementary Table S3**. To visually demonstrate the expression patterns of these differential proteins across different samples, we performed z-score normalization and subsequently constructed a cluster heatmap (**Figure 3C**).

### 3.4 Enrichment analysis of DEPs between control 21 and T21

**Figure 4:**
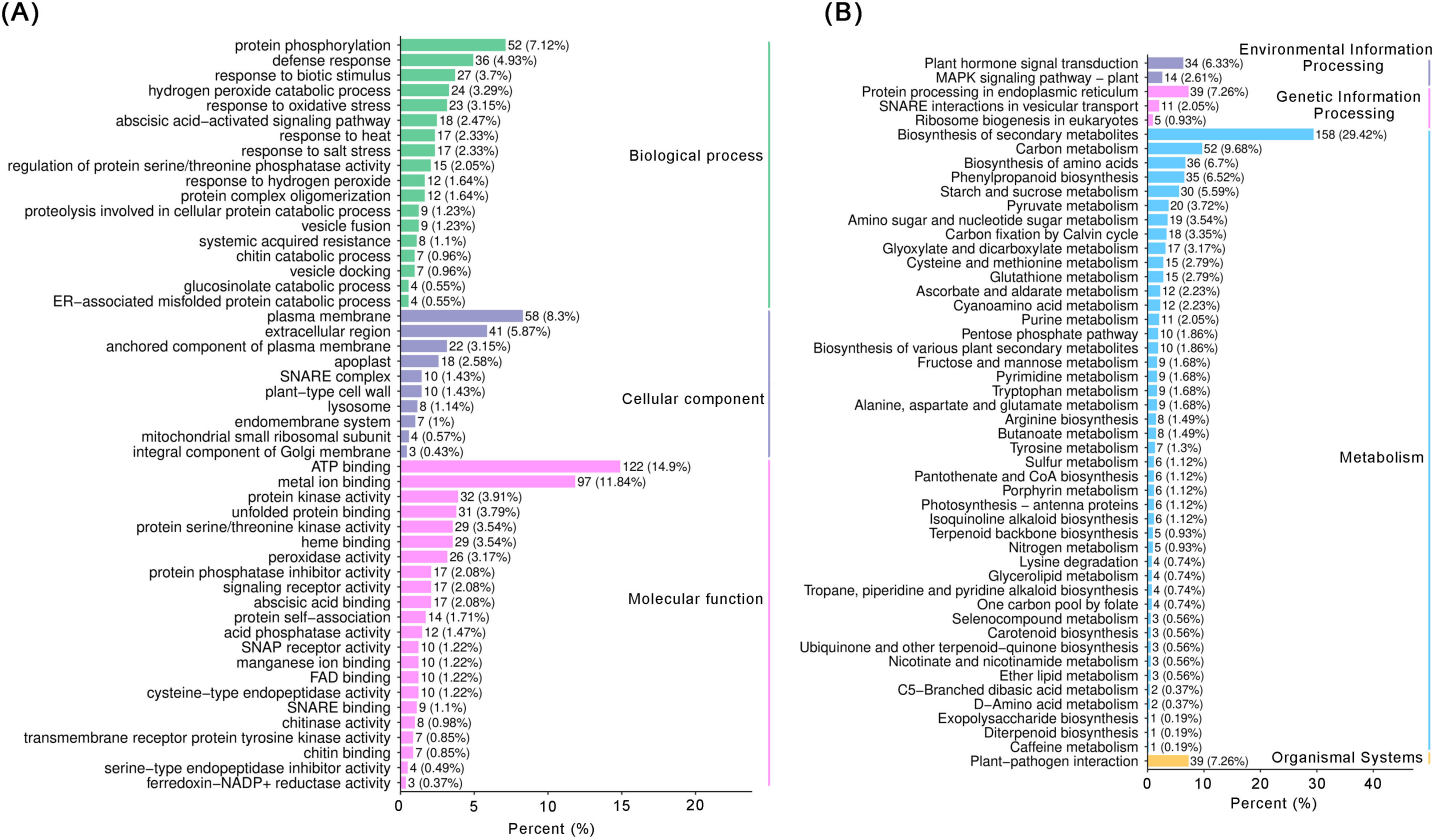
GO and KEGG analysis of the DEPs between control and IBA. **(A)** Bar chart of GO enrichment analysis of DEPs. The horizontal axis represents the proportion of DEPs annotated to the item, and the vertical axis represents the name of the GO item. **(B)** Bar chart of KEGG classification of DEPs. The x-axis represents the proportion of DEPs annotated to the pathway to the total number of annotated proteins, and the y-axis represents the name of the KEGG pathway.

To comprehensively analyze the proteomic changes, we annotated the 1,221 DEPs using GO and KEGG analyses. The GO analysis revealed that the most prominent categories, in order of significance, were protein phosphorylation (accounting for 7.12% of DEPs), translation (5.89%), cytosol (7.87%), ATP binding (14.9%), metal ion binding (11.84%), and structural constituent of ribosome (6.35%) (**Figure 4A**). Subsequently, we conducted a KEGG pathway analysis to explore the core biological pathways enriched by these DEPs. The analysis showed that the majority of the proteins were closely related to the synthesis of secondary metabolites, accounting for 29.42% of the DEPs. Other prominent pathways included biosynthesis of amino acids (6.7%), phenylpropanoid biosynthesis (6.52%), plant hormone signal transduction (6.33%), and starch and sucrose metabolism (5.59%) (**Figure 4B**). In the “Metabolism” biological process category of KEGG, DEPs from a total of 18 metabolic pathways accounted for more than 2% of the total. These pathways encompass 11 metabolic processes, specifically including phenylpropanoid biosynthesis (biosynthesis of secondary metabolites), purine metabolism (nucleotide metabolism), cyanoamino acid metabolism (amino acid metabolism), and eight carbohydrate metabolic processes, namely starch and sucrose metabolism, amino sugar and nucleotide sugar metabolism, ascorbate and aldarate metabolism, pentose and glucuronate interconversions, glycolysis/gluconeogenesis, glyoxylate and dicarboxylate metabolism, pyruvate metabolism, and carbon fixation by Calvin cycle (**Figure 4B**). These metabolic processes play critical roles in plant growth and development.

### 3.5 Metabolomic differences between control 21 and T21

**Figure 5:**
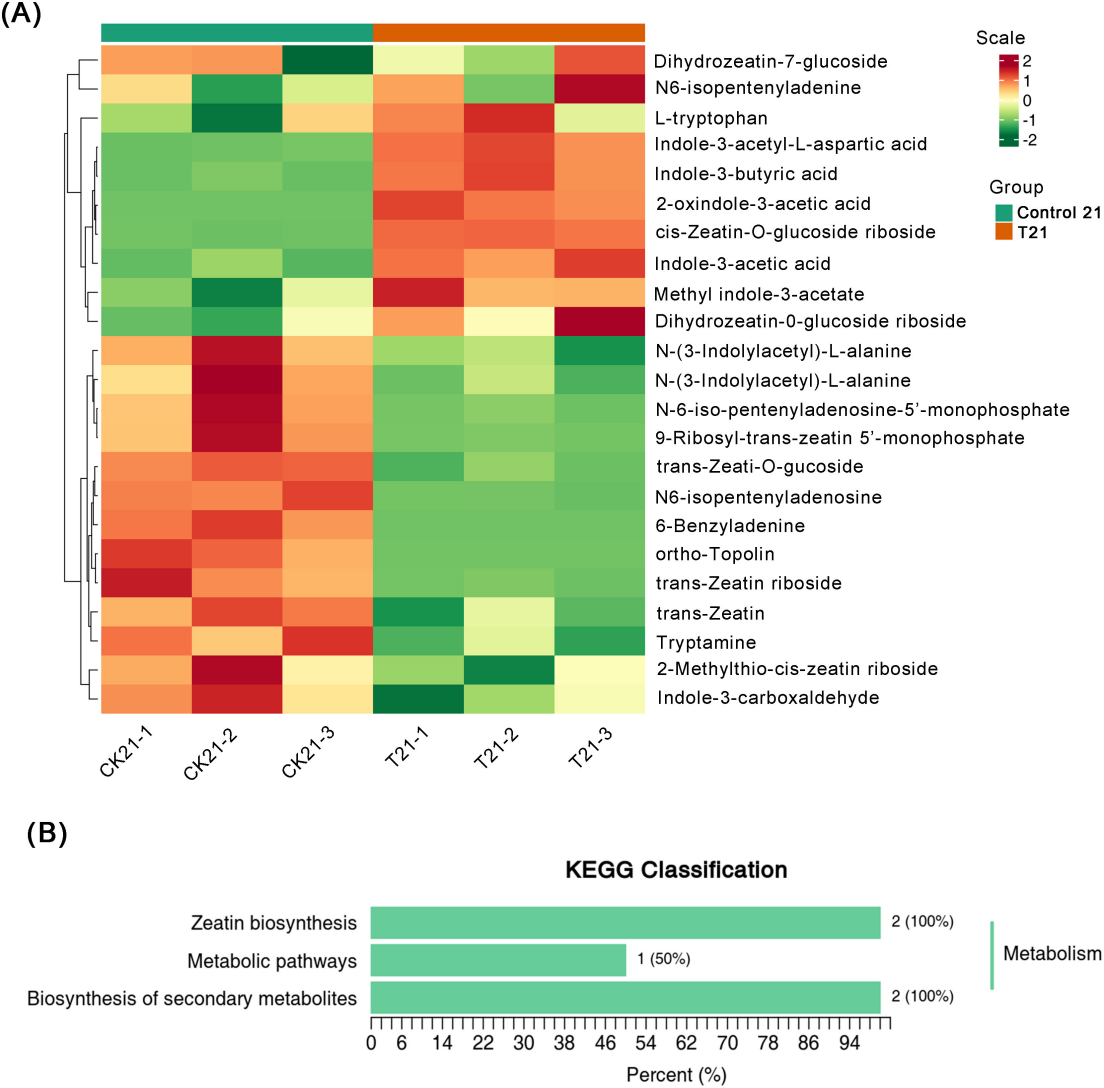
Analysis of differential and specific metabolites following 21 days of IBA treatment in peach rootstocks. **(A)** Heatmap analysis of DEMs between T21 and control 21 groups. **(B)** KEGG classification of DEMs. The vertical axis indicates the names of KEGG metabolic pathways, while the numbers in the figure represent the count of metabolites annotated to each pathway. The numbers in parentheses show the proportion of metabolites annotated to a specific pathway relative to all metabolites annotated within the KEGG database.

To investigate the impact of IBA treatment on metabolites during AR formation in peach rootstocks, we conducted an in-depth metabolomic analysis of the control 21 group and T21 group using LC-MS/MS. Metabolites that exhibited significantly changed (*P* < 0.05) between the CK and IBA treatments were selected for subsequent analysis and summarization. The differential metabolites between control and IBA-treated plants are shown in **Figure 5A**. A total of 10 differential metabolites related to plant hormone signal transduction (*P* < 0.05) were identified. Among them, Indole-3-acetyl-L-aspartic acid, 9-Ribosyl-trans-zeatin 5’-monophosphate, Indole-3-butyric acid, and trans-Zeatin riboside exhibited the most significant differences between control 21 and T21 (**Supplementary Table S4**). Subsequently, KEGG functional annotation and enrichment analysis were performed on the differential metabolites. The results revealed that zeatin biosynthesis, biosynthesis of secondary metabolites, and metabolic pathways were highly enriched (**Figure 5B**).

### 3.6 Multi-omics analysis for screening regulatory pathways

**Figure 6:**
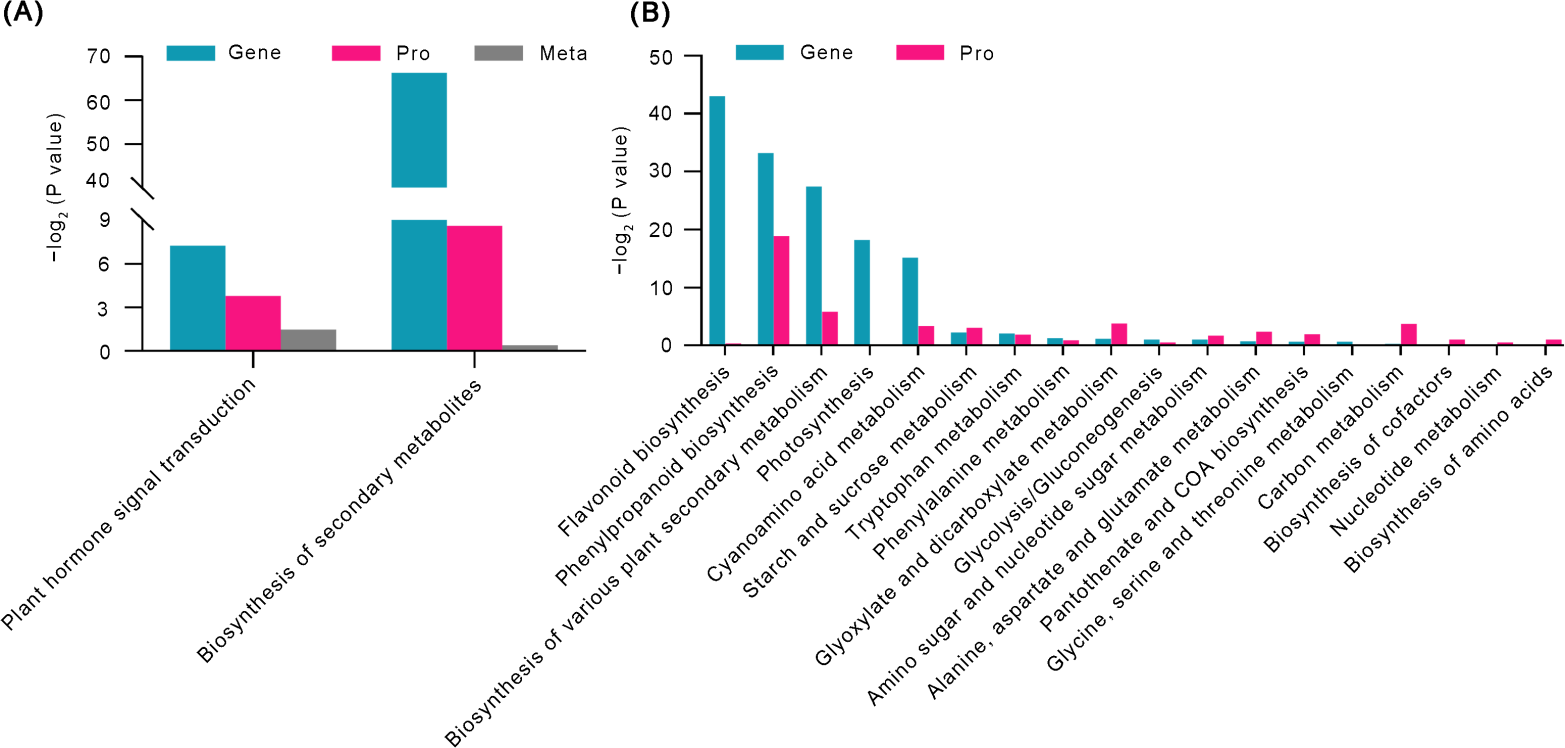
Key pathways involved in IBA-regulated AR formation in peach rootstocks were identified through integrated multi-omics analysis. **(A)** Histogram of KEGG enrichment p-values for the three omics datasets (transcriptome Gene, proteome Pro, and metabolome Meta). **(B)** Significantly enriched KEGG pathways in the transcriptome Gene and proteome Pro. The x-axis represents the enriched pathways.

To elucidate the molecular mechanism underlying IBA-regulated AR formation in peach rootstocks, this study integrated DEGs, proteins, and metabolites by first mapping them to the KEGG pathway database. The analysis aimed to identify commonly enriched pathways across the three datasets and determine the core biochemical and signaling pathways involved. When comparing control 21 and T21 samples, two significantly co-enriched KEGG pathways were identified in all three datasets: plant hormone signal transduction and biosynthesis of secondary metabolites (**Figure 6A**). Meanwhile, we identified significantly enriched biosynthetic pathways for phenylpropanoid biosynthesis in both the transcriptome and proteome (**Figure 6B**). These pathways are critically involved in plant growth, development, and stress responses, indicating their potential role as key regulators in IBA-induced AR formation.

### 3.7 Identification of DEGs through multi-omics analysis

**Figure 7:**
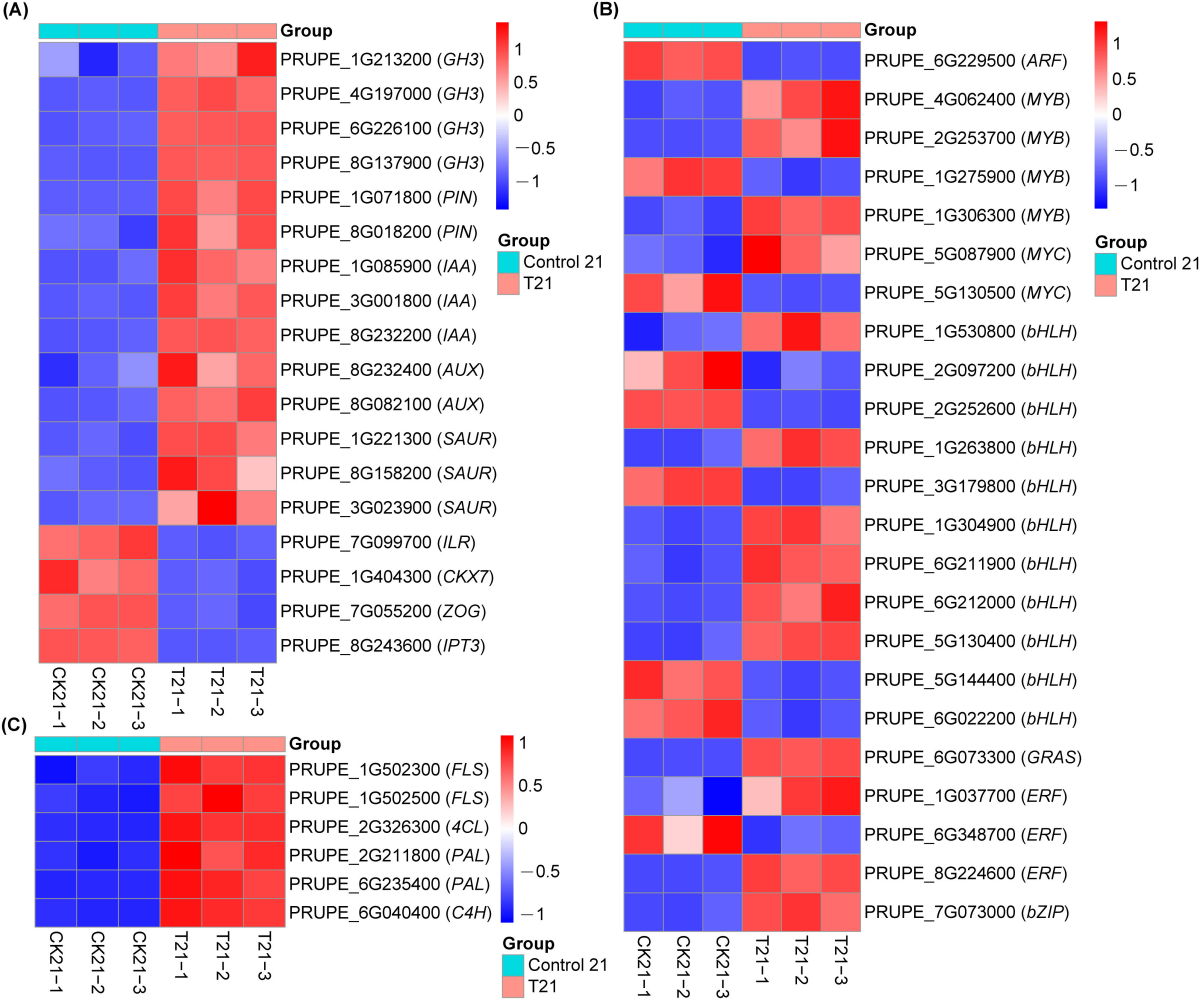
Analysis of DEGs in the control and IBA of plant hormone signal transduction and phenylpropanoid biosynthesis. **(A)** The auxin-related genes differentially regulated by IBA treatment. **(B)** The genes of the transcription factor family related to plant hormones that are differentially regulated by IBA treatment. **(C)** The DEGs related to phenylpropanoid synthesis under IBA treatment. Blue indicates gene down-regulation, while red indicates gene up-regulation.

As shown in **Figure 7**, based on the above KEGG enrichment analysis results, the DEGs in the two most significant pathways were detected (*P* ≤ 0.05). Among them, multiple genes involved in the auxin biosynthesis pathway showed significant expression changes, including four IAA-amide synthetase *GH3* genes (PRUPE_1G213200, PRUPE_4G197000, PRUPE_6G226100, and PRUPE_8G137900), two auxin efflux carrier *PIN* genes (PRUPE_1G071800 and PRUPE_8G018200), and eight auxin response genes *SAURs* and *AUX/IAAs* (PRUPE_1G085900, PRUPE_3G001800, PRUPE_8G232200, PRUPE_8G232400, PRUPE_1G221300, PRUPE_8G158200, PRUPE_3G023900, and PRUPE_8G082100) that were significantly upregulated. Conversely, one IAA-amino acid hydrolase *IAA-Leucine Resistant 1* (*ILR1*) genes (PRUPE_7G099700) were significantly downregulated (**Figure 7A**). The enzymes encoded by the *GH3* gene family catalyze the conjugation of IAA with amino acids, thereby decreasing the concentration of free IAA. Conversely, IAA-amino acid hydrolase cleaves this conjugate to release biologically active IAA (Ludwig-Müller et al., 2005). These results suggest that IBA-induced overexpression of *GH3* genes and suppression of IAA-amino acid hydrolase genes suggest a decrease in free auxin levels and an increase in amino acid-conjugated IAA during AR formation in peach rootstocks.

Previous research has established CTK serves as a negative regulator of AR formation through its inhibitory effect on auxin signaling (Li S. W, et al., 2016). Compared with control group, multiple genes associated with the CTK biosynthesis pathway, including a CTK dehydrogenase *Cytokinin Oxidase/Dehydrogenase 7* (*CKX7)* gene (PRUPE_1G404300), a *Zeatin O-Glucosyltransferase* (*ZOG)* gene (PRUPE_7G055200), and a CTK synthase *IPT3* gene (PRUPE_8G243600), were downregulated in response to IBA treatment (**Figure 7A**). It can be inferred from this that the ARs induced by IBA may be related to the inhibition of CTK synthesis in this study.

Furthermore, to further investigate the role of transcription factors involved in plant hormone pathways in regulating AR formation in peach rootstocks, we analyzed the enriched DEGs that encode transcription factors. These included *ARF* (1), *MYB* (4), Myelocytomatosis (*MYC*) (2), *bHLH* (11), *GRAS* (1), *AP2/ERF* (3), and *bZIP* (1). Previous studies have found that the *AP2/ERF*, *MYB*, *NAC*, *WRKY*, and *bHLH* families are significantly upregulated at the early stages of AR formation in poplar, with the *MYB* and *AP2/ERF* families identified as the most highly regulated transcription factors (Rigal et al., 2012). Additionally, it is known that the *SCR* gene in the GRAS family is involved in meristem maintenance of AR primordia and the initiation of root meristems (Vielba et al., 2011). Our findings indicate that the majority of *bHLH*, *MYB*, *GRAS*, and *bZIP* transcription factors are upregulated, while *ARF* is downregulated during AR formation following IBA treatment (**Figure 7B**). Therefore, our results suggest that different transcription factors play distinct roles in peach rootstocks after 21 days of treatment.

Phenylpropanoid metabolism plays a role in plant development, signal transduction, and stress responses. Evidence has shown that phenylpropanoids are involved in the process of AR (Park et al., 2015; Cheniany and Ganjeali, 2016). Compared with the control group, IBA treatment induced upregulation of one *4-Coumarate-CoA Ligase* (*4CL*) gene, two *Phenylalanine ammonia-lyase* (*PAL*) genes, one *cinnamate-4-hydroxylase* (*C4H*) gene, and two *Flavonol synthase* (*FLS*) genes (**Figure 7C**). This study demonstrates that IBA treatment upregulates the expression of genes related to phenylpropanoid metabolism during AR formation.

### 3.8 qRT-PCR validation of DEGs in the RNA-Seq dataset

**Figure 8:**
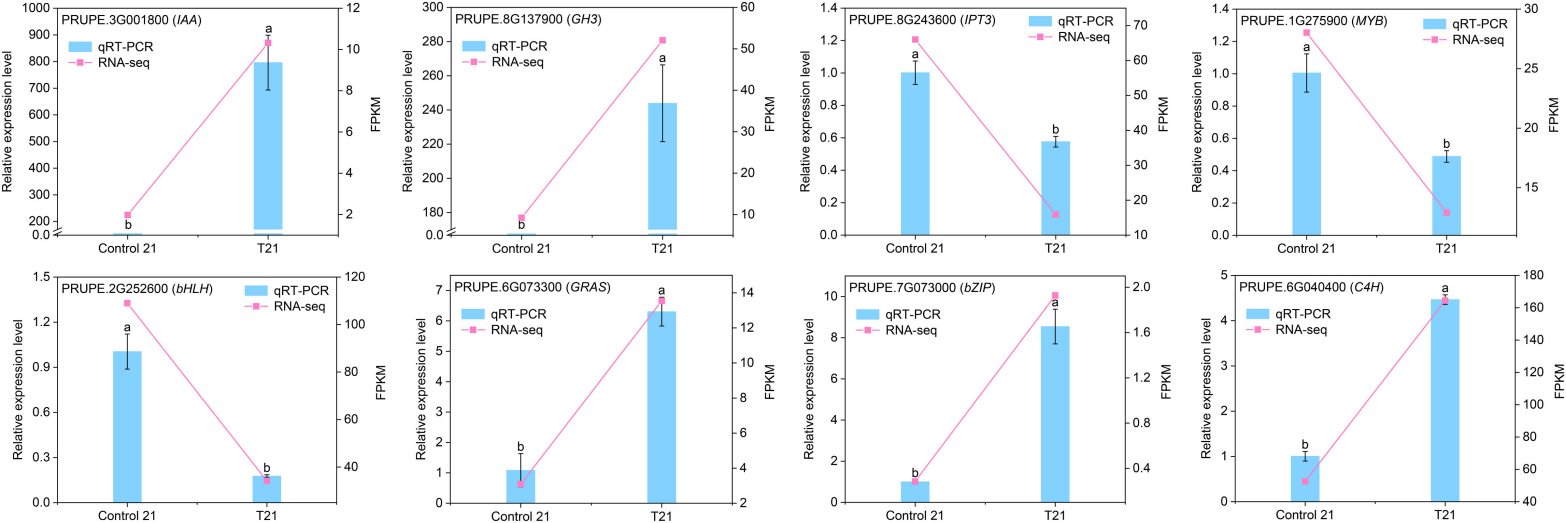
The qRT-PCR results of the expression levels of eight DEGs. *Actin* was used as the reference gene. The left vertical axis represents the qRT-PCR validation data, indicated by blue bar charts. The right vertical axis represents the FPKM values of DEGs in transcriptomic data, shown by pink line graphs.The qRT-PCR data presented are the average values of three biological replicates, with the standard error indicated by the vertical bars. The different lowercase letters in the figure denote statistically significant differences among groups at the *P* < 0.05 level.

Using qRT-PCR, we performed an in-depth analysis of eight DEGs, including two genes involved in auxin synthesis, one gene associated with CTK biosynthesis, four transcription factors, and one gene implicated in phenylpropanoid metabolism. These eight DEGs were selected for qRT-PCR validation. First, they exhibited the most significant expression changes between the control and IBA-treated groups in the RNA-Seq, which helps to effectively validating the accuracy of RNA-Seq. Second, their functional annotations are associated with key pathways identified through multi-omics analysis. The findings revealed that all eight genes detected by qRT-PCR in both control 21 and T21 samples exhibited significant differential expression (*P* < 0.05) (**Figure 8**). Specifically, in the IBA-treated group, the expression levels of *PRUPE_8G137900 (GH3)*, *PRUPE_3G001800 (IAA)*, *PRUPE_6G073300 (GRAS)*, *PRUPE_7G073000 (bZIP)*, and *PRUPE_6G040400 (C4H)* were significantly higher compared to those in the control group, whereas the expression levels of the remaining three genes were elevated in the control group. These results strongly support the reliability of the RNA-Seq data.

## 4 Discussion

The formation of ARs in peach is a complex developmental process orchestrated by intricate interactions between hormones and metabolic pathways. In this study, we employed an integrated multi-omics approach to dissect the molecular mechanisms underpinning IBA-induced AR formation in ‘GF677’ peach rootstock. Previous studies have demonstrated that exogenous IBA can effectively induce the formation of ARs in various plant species (Lei et al., 2018; Wei et al., 2013), yet the precise mechanisms underlying this process remain incompletely understood. Our previous research found 200 mg/L IBA to be the most effective concentration for promoting root formation in peach rootstocks (Zhang et al., 2013; Zhang et al., 2025). The role of IBA in AR formation may involve modulating plant hormones crucial for root development (Druege et al., 2019). Our findings reveal that IBA dynamically alters hormone levels over time, indicating its role in regulating AR formation through modifying endogenous IAA and CTK concentrations in peach rootstocks. It is well established that the balance and interaction between auxin and CTK are critical for post-embryonic organogenesis, which can be disrupted by wounding due to its stimulatory effect on the biosynthesis of these hormones (Ikeyama et al., 2010; Ikeuchi et al., 2019). Furthermore, studies have demonstrated that the vascular cambium, which is crucial for AR formation, relies on the cooperative effects of auxin and CTK, as well as their intricate cross-regulation of signaling pathways (Wang et al., 2021). In this study, the highest rooting rate and IAA/CTK ratio were observed at 21 days following treatment with 200 mg/L IBA, compared to the control group (**Figure 1**). Consequently, we identified 21 days post-treatment with 200 mg/L IBA as the crucial time for AR formation in ‘GF677’ peach rootstock. At this time point, transcriptome, proteome, and targeted metabolome analyses were performed to uncover the underlying molecular regulatory mechanisms.

Phytohormone signaling pathways exhibited consistency and significant enrichment across multi-omics analyses (**Figures 2**, **4**, **5**). However, IBA treatment triggered complex hormonal crosstalk rather than merely altering auxin concentrations. The KEGG pathway analysis revealed that the majority of proteins were significantly enriched in pathways such as amino acid biosynthesis, phenylpropanoid biosynthesis, starch and sucrose metabolism, and plant hormone signal transduction. These findings were consistent with the RNA-Seq data. Subsequently, we analyzed the DEGs in the two most significant pathways (*P* ≤ 0.05). Auxin, a major plant hormone, plays a pivotal role in promoting AR formation, and its accumulation is associated with enhanced rooting ability. For example, in eucalyptus, higher levels of IAA were found in juvenile cuttings that root easily compared to mature cuttings that are difficult to root (Abu-Abied et al., 2012). The increase in IAA content observed in the phenotypic analysis was associated with the identification of numerous proteins involved in IAA homeostasis and polar transport in our dataset. GH3 is responsible for converting IAA into inactive forms (Staswick et al., 2005; Yang et al., 2008), whereas ILR1 contributes to the hydrolysis of IAA conjugates in plant cells, thereby activating auxin signaling (Sanchez Carranza et al., 2016). In our data, four *GH3* genes were significantly upregulated, whereas the *ILR1* gene (PRUPE_7G099700) was suppressed. The enzymes encoded by *GH3* genes conjugate free IAA to amino acids, forming compounds such as IAA-aspartate, thereby reducing the concentration of free IAA (Luo et al., 2023). In contrast, the ILR1 hydrolase catalyzes the hydrolysis of these conjugates, releasing physiologically active IAA. This suggests that excessively high initial auxin levels during AR induction may inhibit root primordia initiation. Meanwhile, the interaction between GH3 and ILR1 enzymes ensures a sustained, low-intensity auxin signal required in the later stages of AR formation, which is crucial for the continuous development of root primordia. Members of the PIN protein family are essential for auxin efflux (da Costa et al., 2013). Additionally, other DEGs, such as *AUX/IAA* and *SAUR*, also regulate the formation of ARs through IAA signal transduction. Zeatin riboside (ZR), the predominant transport form of CTKs, promotes cell division, differentiation, and AR formation (Alvarez et al., 2020; Kuroha et al., 2002). Genes related to the CTK biosynthesis pathway, such as *CKX7*, *ZOG*, and *IPT3*, exhibited low expression levels in the RNA-Seq. The initiation of root primordia development was triggered by a shift in the IAA/CTK ratio toward auxin, which is supported by the established understanding that CTK is a potent inhibitor of AR formation (Mao et al., 2019). Relevant studies have shown that 345 transcription factor genes are regulated by IBA and contribute to AR formation in mung bean seedlings (Li S. W, et al., 2016). When analyzing the DEGs involved in plant hormone pathways, we observed that various transcription factors, including *ARF*, *MYB*, *MYC*, *bHLH*, *GRAS*, *AP2/ERF*, and *bZIP*, were enriched in these pathways. Notably, during AR formation under IBA treatment, most *bHLH*, *MYB*, *GRAS*, and *bZIP* transcription factors were upregulated. The downregulation of *ARF* may be mediated by the inhibition of their activity by Aux/IAA proteins, a process facilitated by the reduction in IBA concentration within the auxin signaling pathway (Lanctot and Nemhauser, 2020). Consequently, our results suggest that different transcription factors may play specific roles in regulating the hormonal signaling pathways of peach rootstocks treated for 21 days. Additionally, in this study, both auxin and zeatin exhibited significant changes in metabolomic analysis. Therefore, proteins related to IAA and CTK signaling may mediate the regulation of AR formation in peach rootstock samples treated with IBA.

Based on the proteomic research findings, we have identified that the majority of proteins are closely associated with the synthesis of secondary metabolites, accounting for 29.42% of DEPs. Specifically, phenylpropanoid biosynthesis constitutes 6.52% of this proportion. Within this pathway, 4CL serves as a pivotal enzyme in the biosynthesis of monolignols and flavonoids in phenylpropanoid metabolism and plays a role in lignin synthesis in poplar (Cao et al., 2020; Sun et al., 2013). Simultaneously, PAL contributes to lignin synthesis by catalyzing the conversion of phenylalanine into precursors of lignin monomers (Gho et al., 2020). Furthermore, C4H and FLS are equally crucial enzymes in the phenylpropanoid biosynthesis pathway (Kumar et al., 2016; Hartmann et al., 2005). In the RNA-Seq analysis of this study, we observed upregulated expression levels of one *4CL* gene, two *PAL* genes, one *C4H* gene, and two *FLS* genes. Phenylpropanoids act as precursors to lignin, a polymer essential for the structural integrity of newly formed vascular tissues, which function to integrate the primordium with the main stem system (Dwivedi et al., 2024). This finding indicates that IBA may enhance lignin synthesis and improve root elasticity in peach rootstocks. Furthermore, under IBA treatment, various phenolic compounds are synthesized and secreted into the rhizospheric environment by root systems via phenylpropanoid biosynthetic genes *4CL*, *PAL*, *C4H*, *FLS*, where they promote nutrient uptake and nitrogen fixation. Concurrently, carbon source allocation and utilization are regulated by the expression and activity of these genes, as the phenylpropanoid pathway is closely linked with central carbon metabolism (Hura et al., 2025). This process supplies the necessary energy and material foundation for the growth and development of ‘GF677’ root systems. Indeed, previous studies have demonstrated a positive correlation between the expression levels of phenylpropanoid biosynthesis pathway components and lignin content in *Toona sinensis* (Sui et al., 2019). Additionally, increased expression of genes involved in phenylpropanoid biosynthesis has been shown to promote root growth in tobacco plants (Park et al., 2020). The metabolomics data further support these conclusions, revealing that IBA treatment in peach rootstocks significantly enriches the pathways related to secondary metabolite biosynthesis. In the metabolome, 6-benzyladenine (6-BA) was identified as a significant differential metabolite (**Figure 5A**). As a synthetic CTK not applied in this experiment, its presence was anomalous. It could potentially be a misannotation of a structurally similar endogenous metabolite; however, its marked accumulation suggested that CTK composition was significantly regulated during IBA-induced adventitious root formation. Future studies are required to conclusively confirm the identity of this metabolite using standard compounds and separation techniques.

In summary, this study reveals the regulatory mechanism through which IBA promotes adventitious root formation in peach rootstocks: Under exogenous IBA treatment, free IAA is conjugated and internal rooting inhibition thresholds are reduced through the upregulation of *GH3* genes, which leads to the coordinated suppression of the CTK biosynthesis pathway. These changes result in an increased IAA/CTK ratio, which subsequently activates key transcription factors *bHLH*, *MYB*, and *AP2/ERF*, ultimately driving the expression of downstream proteins and metabolites. Concurrently, the phenylpropanoid biosynthesis pathway is strongly induced, providing essential lignin required for nascent root primordia and facilitating the occurrence of ARs. Studies have shown that IBA treatment promotes the formation of ARs in peach clonal rootstocks by improving plant hormone levels and phenylpropane biosynthesis.

## 5 Conclusion

In this study, we demonstrate that treatment with 200 mg/L IBA for 21 days significantly increased the rooting rate and IAA/CTK ratio, a key driver of rooting competence. Integrated transcriptomic, proteomic, and metabolomic analyses consistently highlighted two central pathways: plant hormone signal transduction and phenylpropanoid biosynthesis. IBA promoted a shift toward conjugated auxin forms by upregulating *GH3* and downregulating *ILR1*, while concurrently suppressing CTK synthesis through reduced expression of *IPT3* and *CKX7*. Additionally, key genes involved in phenylpropanoid biosynthesis (*PAL*, *C4H*, *FLS*) were upregulated. Several transcription factor families (*bHLH*, *MYB*, *GRAS*, *bZIP*, *AP2/ERF*) were activated, further integrating hormonal signals to orchestrate rooting. These results provide a comprehensive molecular framework for understanding AR formation in peach rootstocks and offer practical strategies for improving propagation in woody plants.

## Data Availability

The RNA-Seq raw data from this study have been deposited in the National Center for Biotechnology Information (NCBI) (https://www.ncbi.nlm.nih.gov/) Sequence Read Archive (SRA) under the accession number PRJNA1327572.
