# Supplementary material for: Multi-omics analysis reveals the mechanism of adventitious roots formation in peach green branch cuttings
Source: Front Plant Sci. 2026 Jan 5;16:1740503. doi: 10.3389/fpls.2025.1740503 (PMC12812935; doi:10.3389/fpls.2025.1740503)
Supplement: Supplementary file 1 [file DataSheet1.docx]

Supplementary Material

# Supplementary Figures and Tables

## Supplementary Figures


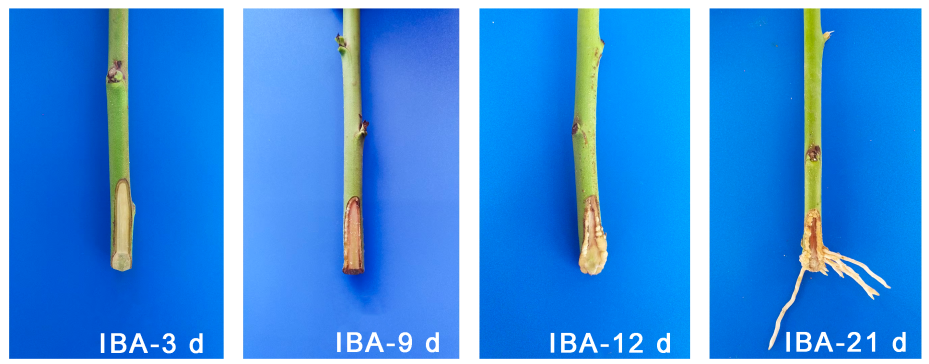


**Supplementary Figure S1.** The rooting phenotypes of the GF677 rootstock after IBA treatment on the 3rd, 9th, 12th, and 21st days after cutting.


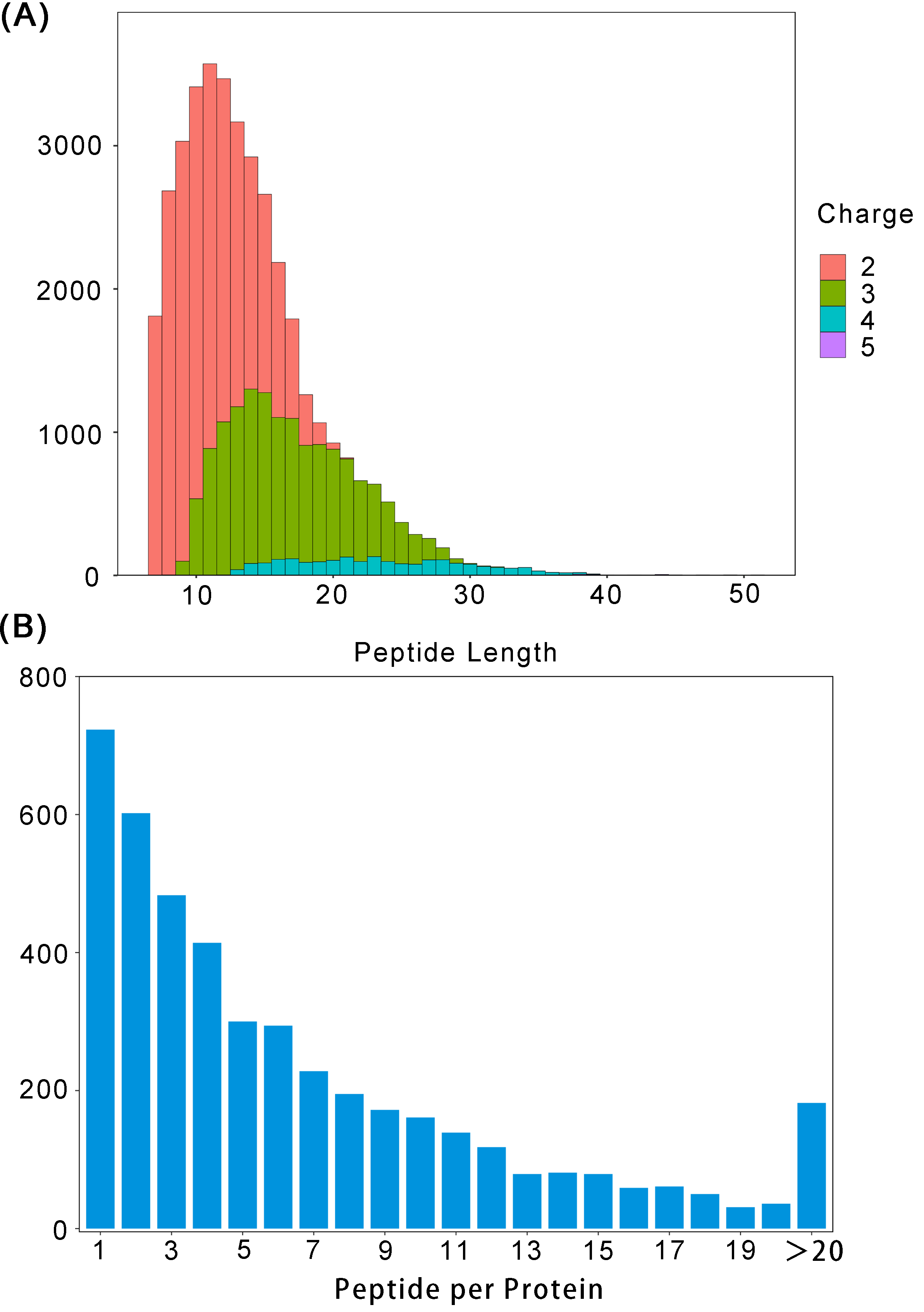


**Supplementary Figure S2.** Qualitative result quality assessment. The data obtained from mass spectrometry undergoes a series of quality control evaluations following the completion of database searching. **(A)** The detection of peptide length distribution. **(B)** The relative distribution of peptide segments in different samples.

**1.2** **Supplementary Tables**

**Supplementary Table S1. Primer sequences for qRT-PCR**

| Gene ID | Forward primer | Reverse primer |
| --- | --- | --- |
| *Actin* | 5'-GATTCCGGTGCCCAGAAGT-3' | 5'-CCAGCAGCTTCCATTCCAA-3' |
| PRUPE_8G137900 (*GH3*) | 5'-GGGCGTCTATCAACCAGTATAAG-3' | 5'-ATGCGGTAAAGATGGGCTAAA-3' |
| PRUPE_3G001800 (*IAA*) | 5'-CAGGCTTGGGCTTAGCTTAT-3' | 5'-CTCCTTCGGCTGGTTTGAATA-3' |
| PRUPE_8G243600 (*IPT3*) | 5'- GTGGAATTTGCATCCGTTGG-3' | 5'- TGAGCACTTGGCCCATAAA-3' |
| PRUPE_1G275900 (*MYB*) | 5'- GCATGGTGGTGATCCTGAAA-3' | 5'-CTGCTTCTCTTCCTCCCAATAAC-3' |
| PRUPE_2G252600 (*bHLH*) | 5'-CTGAAGGTGCTGATGAAGAAGA-3' | 5'-TCTTGTCGGACTTGGGAATTAG-3' |
| PRUPE_6G073300 (*GRAS*) | 5'- GGCAGTAACCACAGCAGTAA-3' | 5'-GCAGGAGATTCAGTAGCAGAAA-3' |
| PRUPE_7G073000 (*bZIP*) | 5'- CCAGCAGTTAGAGACAAGTAGG-3' | 5'-CTCCTACAATACCTCCTCCAAAG-3' |
| PRUPE_6G040400 (*C4H*) | 5'-GGAAGAAGCCTGAGGAGTTTAG-3' | 5'-ACAGCTTCTTCTCCCAACAC-3' |

**Supplementary Table S2. Summary of RNA sequencing data**

| Sample | Group | Raw Reads | Clean Reads | Clean Base (G) | Error Rate (%) | Q20 (%) | Q30 (%) | GC Content (%) |
| --- | --- | --- | --- | --- | --- | --- | --- | --- |
| CK21-1 | control 21 | 60,125,958 | 56,908,802 | 8.54 | 0.01 | 99.04 | 97.01 | 45.12 |
| CK21-2 | control 21 | 56,388,294 | 53,797,452 | 8.07 | 0.01 | 99.11 | 97.23 | 45.05 |
| CK21-3 | control 21 | 59,253,236 | 56,054,598 | 8.41 | 0.01 | 99.07 | 97.12 | 45.13 |
| T21-1 | T21 | 73,991,746 | 70,259,306 | 10.54 | 0.01 | 99.1 | 97.19 | 45.38 |
| T21-2 | T21 | 71,762,706 | 68,274,264 | 10.24 | 0.01 | 99.06 | 97.09 | 45.38 |
| T21-3 | T21 | 61,593,588 | 58,396,846 | 8.76 | 0.01 | 99.03 | 96.99 | 45.15 |

**Supplementary Table S4. Screening results of differential metabolites.**

| Index | Compounds | Class | T21-1 | T21-2 | T21-3 | CK21-1 (control 21) | CK21-2 (control 21) | CK21-3 (control 21) | p-value | FDR | Fold change | Log_2_FC | Type |
| --- | --- | --- | --- | --- | --- | --- | --- | --- | --- | --- | --- | --- | --- |
| IAA-Asp | Indole-3-acetyl-L-aspartic acid | Auxin | 12.2077019 | 13.0967622 | 11.6001034 | 4.93690812 | 5.05265298 | 5.18692187 | 0.002906622 | 0.011142053 | 2.431694324 | 1.281961886 | up |
| tZRMP | 9-Ribosyl-trans-zeatin 5'-monophosphate | CTK | 16.6945414 | 17.2697838 | 16.521055 | 34.9146182 | 48.4902927 | 38.2970872 | 0.027976827 | 0.041554527 | 0.414827866 | -1.269415284 | down |
| IBA | Indole-3-butyric acid | Auxin | 15.7658774 | 17.417393899999997 | 14.925802800000001 | 4.40280576 | 5.09387071 | 4.390129900000001 | 0.002029694097906469 | 0.011142052677167317 | 3.464372787967375 | 1.7925941813390174 | up |
| tZR | trans-Zeatin riboside | CTK | 10.160842500000001 | 10.763338000000001 | 9.91408097 | 30.2798207 | 24.9912838 | 23.067235800000002 | 0.016979467778223654 | 0.032543979908262 | 0.3936547717491023 | -1.3449971286986577 | down |
| iPRMP | N-6-iso-pentenyladenosine-5'-monophosphate | CTK | 10.3094716 | 11.839399 | 9.74670016 | 29.9637316 | 45.139001799999996 | 33.0176146 | 0.02980023562977076 | 0.0415545267355359 | 0.29500062985368863 | -1.761210060121958 | down |
| cZROG | cis-Zeatin-O-glucoside riboside | CTK | 12.3379362 | 12.430310400000002 | 12.0235422 | 3.10684062 | 2.94518703 | 3.05035918 | 2.671952648415905e-5 | 6.145491091356581e-4 | 4.041993543796688 | 2.015067017328747 | up |
| IPR | N6-isopentenyladenosine | CTK | 1.51843719 | 1.50779948 | 1.38526891 | 5.12102055 | 5.04589347 | 5.7719445 | 0.0027644053972243083 | 0.011142052677167317 | 0.2767767575365867 | -1.8532052978469338 | down |
| OxIAA | 2-oxindole-3-acetic acid | Auxin | 37.6233799 | 32.8106406 | 30.7417258 | 0 | 0 | 0 | 0.003633681 | 0.011939237 | Inf | Inf | up |
| oT | ortho-Topolin | CTK | 0 | 0 | 0 | 0.254896232 | 0.227599106 | 0.1813457 | 0.00927978 | 0.023714994 | 0 | -Inf | down |
| BAP | 6-Benzyladenine | CTK | 0 | 0 | 0 | 0.223980959 | 0.261958731 | 0.204299597 | 0.005365733 | 0.015426484 | 0 | -Inf | down |
